# Optimized Extraction of Phenylpropanoids and Flavonoids from Lemon Verbena Leaves by Supercritical Fluid System Using Response Surface Methodology

**DOI:** 10.3390/foods9070931

**Published:** 2020-07-14

**Authors:** Francisco Javier Leyva-Jiménez, Jesús Lozano-Sánchez, Álvaro Fernández-Ochoa, María de la Luz Cádiz-Gurrea, David Arráez-Román, Antonio Segura-Carretero

**Affiliations:** 1 Functional Food Research and Development Center, Health Science Technological Park, Avenida del Conocimiento s/n, E-18016 Granada, Spain; jleyva@cidaf.es (F.J.L.-J.); alvaroferochoa@ugr.es (Á.F.-O.); darraez@ugr.es (D.A.-R.); ansegura@ugr.es (A.S.-C.); 2 Department of Food Science and Nutrition, University of Granada, Campus of Cartuja, 18071 Granada, Spain; 3 Department of Analytical Chemistry, Faculty of Sciences, University of Granada, Fuentenueva s/n, E-18071 Granada, Spain

**Keywords:** *Lippia citriodora*, supercritical fluid extraction, HPLC-ESI-TOF-MS, central composite design, response surface methodology

## Abstract

The aim of this work was to optimize the recovery of phenolic compounds from *Lippia citriodora* using supercritical fluid extraction (SFE). To achieve this goal, response surface methodology based on a 2^3^ central composite design was used to evaluate the effects of the following experimental factors: temperature, pressure and co-solvent percentage. The effects of these variables on the extraction yield and total polar compound contents were evaluated. With respect to the phytochemical composition, an exhaustive individual phenolic compound quantitation was carried out by HPLC-ESI-TOF-MS to analyze the functional ingredients produced by this system design. To the best of our knowledge, this is the first time that a standardized supercritical fluid process has been developed to obtain functional ingredients based on phenolic compounds from *L. citriodora* in which the individual compound concentration was monitored over the different SFE conditions. The results enabled the establishment of the optimal technical parameters for developing functional ingredients and revealed the main factors that should be included in the extraction process control. This functional food ingredient design could be used as a control system to be applied in nutraceutical and functional food production industry.

## 1. Introduction

In recent decades, the prevalence of several diseases such as obesity and diabetes has increased, causing high morbidity and premature mortality and greatly increasing health costs. Consequently, researchers have focused their investigations on the study of alternative treatments to prevent these diseases and to improve human health. In this sense, botanical sources have been claimed to provide beneficial properties related to several physiological disorders. For these reasons, in the last few years, researchers have focused on the challenge of the extraction of bioactive compounds from plants in order to increase the nutritional value of food products [1]. However, the amounts of compounds that are contained in the natural sources, even after some extraction procedures, are usually less than those needed to provide beneficial effects in the organism. Furthermore, different aspects may determine the retrieval of these bioactive compounds. Hence, it is necessary to evaluate the extraction method and the influence of each extraction parameter to discern the effectiveness of the process in terms of extract composition and extraction yield, since both variables can have a great influence in the balance of economic costs and profits.

Advanced extraction technologies have been claimed to be efficient alternatives when compared with conventional extraction methods in terms of their solvents (typically using GRAS (generally recognized as safe) solvents), time consumption, selective extraction and costs, enabling their utilization in developing functional food ingredients. All these characteristics result in a decreased environmental impact; thus, they are considered to be environmentally friendly techniques [2,3]. Indeed, supercritical fluid extraction (SFE) has been receiving growing interest as a sustainable technology which can be used without organic solvents [4] to revalorize food by-products [5,6] or extract specific phytochemicals [7,8]. SFE is an extraction process where analytes are dissolved in a supercritical fluid, which could be considered either as expanded liquid or compressed gas. Thus, it has liquid-like solvent power and gas-like diffusivity, giving it special properties that are useful in the recovery of valuable compounds and enabling a selective extraction of compounds through modifying the density and viscosity of the solvents used [9]. The most used supercritical fluid is CO_2_ since it is a nontoxic, nonflammable and cheap solvent. CO_2_ has a critical point of 73.8 bar and 31.1 °C, allowing the experiments to be run at room temperature; this is in contrast with other solvents such as water (218.3 bar and 347 °C) or methanol (79.8 bar and 239 °C). In addition, it is easily removable from the extract under atmospheric conditions. It is widely used to extract lipophilic compounds since CO_2_, due to its structure, is a nonpolar solvent that has a low capability of dissolving polar compounds. In order to recover more polar compounds such as flavonoids, procyanidins, purines or hydrophilic vitamins, CO_2_ is commonly used in combination with polar GRAS modifiers (i.e., ethanol) which would serve to enhance the extraction of polar compounds based on the formation of hydrogen bonds and the dipole–dipole or dipole–induced-dipole interactions between co-solvent and the analytes [10]. According to several reports, the addition of co-solvent may positively affect the extraction of phenolic compounds [11,12,13]. Consequently, SFE can be applied to recover phenolic compounds from botanical sources.

*Lippia citriodora*, commonly known as lemon verbena, is a plant that is native to South America and has traditionally been used to alleviate fever or stomachache. Recent studies have also attributed anti-inflammatory, antibacterial and anti-obesogenic properties to several phytochemicals in its composition, such as verbascoside or luteolin 7-diglucuronide [14,15,16], revealing this plant to be an interesting natural source of functional ingredients for the treatment of several disorders. Although the use of other advanced extraction methods to extract phytochemicals of lemon verbena has been previously reported [17,18], no research has focused on the recovery of polar compounds of lemon verbena by SFE. Therefore, the development of an SFE process to recover bioactive compounds from lemon verbena would be of interest to producers, as such compounds could be used in developing functional food.

The purpose of this work was to develop, for the first time, an SFE process to optimize the retrieval of bioactive constituents from lemon verbena leaves; the bioactive constituents considered are mainly phenylpropanoids and flavonoids and may be used as functional ingredients. To achieve that goal, response surface methodology (RSM) based on a 2^3^ central composite design (CCD) model to evaluate the effect of independent factors on the recovery of bioactive phenols was applied. The combination of this extraction system with an analytical HPLC-ESI-TOF-MS platform was used to determine the extraction variables which have relevant effects on the extraction procedures so that the recovery of bioactive compounds from lemon verbena leaves can be maximized.

## 2. Materials and Methods

### 2.1. Reagents

All reagents used in this work were at least of analytical reagent grade and used as received. SFE experiments were performed using CO_2_ and ethanol as modifier. Solvents were purchased from Air Product and Chemicals (Allentown, PA, USA) and VWR chemicals (Radnor, PA, USA). Ottawa sand was provided by Fisher Scientific (Leicestershire, UK). Glass wool was acquired from Sigma-Aldrich (Steinhemin, Germany). LC-MS-grade acetonitrile was purchased from Fisher chemicals (Waltham, MA, USA) and double-deionized water (conductivity of <18.0 MΩ) was obtained using a Milli-Q system acquired from Millipore (Bedford, MA, USA). Formic acid was purchased from Sigma-Aldrich (Steinheim, Germany). Apigenin, loganic acid, kaempferol 3-glucoside, quercetin, and verbascoside were purchased either from Fluka, Sigma-Aldrich (Steinheim, Germany) or Extrasynthese (Genay Cedex, France).

### 2.2. Plant Material

Lemon verbena leaves were provided by Monteloeder (Alicante, Spain). Leaves were milled using a ZM200 ultracentrifugal grinder (Retsch GmbH, Haan, Germany) with a 12-tooth rotor and 1-mm mesh sieve. The material was kept at room temperature and protected from light until extraction.

### 2.3. Supercritical Extraction Procedures

SFE was carried out with a Waters Prep SFE 100 Supercritical Fluid Extraction System (Waters, TharSFC, Thar Technologies Inc., Pittsburgh, PA, USA) equipped with CO_2_ and co-solvent pumps (model P-50), an automated back pressure regulator, low and high pressure heating exchangers, a pressurized extraction vessel (100 mL) and pressurized collection vessels. The SFE system was connected to an Accel 500 LC chiller produced by Thermo Scientific (TharSFC, Thar Technologies, Inc., Pittsburgh, PA, USA). Ground leaves were introduced into the extraction vessel by mixing 10 g of sample with 20 g of Ottawa sand and loaded this mixture into the stainless steel extraction cell. In order to prevent sample projection, glass wool was added to the top and the bottom of the extraction vessel. Experiments were conducted with the use of the Thar Instruments Process Suit software (Waters), selecting the following technical parameters: dynamic mode total time of 90 min and total solvent mass flow rate of 30 g/min. Since different percentages of co-solvent were used to optimize the extraction process, the CO_2_ and co-solvent mass flow rates over the different runs were introduced in the software according to the experimental design described below. As a result, the CO_2_ and co-solvent mass flow rates used were from 24.9 to 28.5 g/min and from 1.5 to 5.1 g/min, respectively. After the extraction process, co-solvent was immediately evaporated using a rotary evaporator (Buchi R210, Flawil, Switzerland), and dried extracts were stored at −20 °C until HPLC analysis.

### 2.4. Design of Experiments

Response surface methodology (RSM) was used to evaluate the effect of SFE parameters on retrieval and yield of phenolic compounds. For this purpose, a CCD model with two axes, four central points and three levels (−1, 0, 1) for each independent variable was applied: (a) temperature, 40, 50 and 60 °C; (b) pressure, 150, 275 and 400 bar; and (c) co-solvent percentage, 7, 11 and 15%. This model procured a total of 18 experiments which were conducted in a randomized order (Table 1). Finally, the bioactive compound contents in extracts as determined by HPLC-ESI-TOF-MS and the extraction yield were response variables. The extraction yield of each procedure was calculated considering the weight of dried extract and the amounts of lemon verbena used in the procedure (Equation (1)):

(1) $$Yield\;\left(\%\right)=\frac{Weight\;of\;dried\;extract\left(g\right)}{Weight\;of\;dried\;leaves\;used\;\left(g\right)}\times 100$$

The obtained results were statistically processed using Statgraphics Centurion software XVI provided by Statpoint Technologies (Warrenton, VA, USA). Four different statistical parameters were used to evaluate the model fitting: coefficient of determination (R^2^), coefficient of variation (CV), lack-of-fit test and model value [18].

Responses were fitted to the second-order polynomial model summarized in Equation (2):

(2) $$Y=\beta_{0}+\overset{n}{\underset{\;i\;=1}{\sum }}\beta_{i}Z_{i}+\overset{n}{\underset{i=1\;}{\sum }}\beta_{\mathrm{ii}}{\;Z}_{i}^{2}+\overset{n}{\underset{i=1}{\sum }}\overset{n}{\underset{j=i+1}{\sum }}\beta_{\mathrm{ij}}{\;Z}_{i}{\;Z}_{j}$$

where *n* is the number of variables; β_0_ is a constant that fixes the response at the central point of the experiments; and β*_i_*¸ β*_ii_* and β*_ij_* are the coefficients of the linear, quadratic and interaction parameters, respectively. The parameters Z*_i_* and Z*_j_* represent the values of independent variables, and, finally, Y is the value of each response.

ANOVA tests were performed to evaluate the statistical significance of each independent factor (*p-*values).

### 2.5. Identification of Polar Profile of SFE Lemon Verbena Extracts by HPLC-ESI-TOF-MS

Dried extracts were reconstituted using absolute ethanol until a concentration at 2500 µg/mL and filtered through a 0.2 μm filter before analysis. An RRLC 1200 system was used to analyze the extracts (Agilent Technologies, Palo Alto, CA, USA). This analytical platform was equipped with a vacuum degasser, an automated sampler, a binary solvent delivery system and a UV-Vis detector. The RRLC system was coupled to a Bruker micrOTOF mass spectrometer (Bruker Daltonik, Bremen, Germany) using an electrospray interface (ESI) (model G1607 from Agilent Technologies, Palo Alto, CA, USA).

Phytochemical separation was accomplished using a 150 mm × 4.6 mm id, 1.8 µm particle diameter Zorbax Eclipse Plus C18 column (Agilent Technologies, Palo Alto, CA, USA). The eluents were water:acetonitrile 90:10 (v/v) with 0.1% formic acid (eluent A) and acetonitrile (eluent B). The injection volume was 10 µL, and the chromatographic separation was carried out according to a multistep gradient [17] in a total run time of 35 min at room temperature. The flow rate was 0.5 mL/min.

The effluent from the HPLC system was introduced into the mass spectrometer after reducing the flow rate with a “T”-type splitter, achieving a flow rate of less than 0.2 mL/min and ensuring correct ionization by ESI. All MS assays were developed in negative ion mode and considering a mass range from 50 to 1000 *m/z*. The transfer and source parameters (capillary voltage, drying gas temperature, drying gas flow and nebulizing pressure) were set according to the literature [18]. It is necessary to remark that each analysis was externally calibrated at the beginning of each MS experiment with a sodium formate cluster injected by a 74900-00-05 Cole Palmer syringe pump (Vernon Hills, IL, USA). Moreover, mass spectra provided by MS equipment were calibrated before phytochemical characterization. This identification was accomplished with the use of Data Analysis 4.0 software (Bruker Daltonics, Billerica, MA, USA), whose sophisticated CHNO algorithm supported a good identification.

Four commercial standards (loganic acid, quercetin, verbascoside and kaempferol 3-glucoside) were used to quantify phenolics in samples. Calibration curves were performed with eleven points at different concentrations (from 0.5 to 150 µg/mL), and apigenin (25 µg/mL) was used as internal standard. The linearity of all calibration curves was above 0.99. Quantitation of phytochemicals was calculated by plotting the standard concentration as a function of the peak area (standard area/ internal standard area) and interpolating in the corresponding calibration curve. Total phytochemical family and total phenolic content in leaf extracts were tentatively calculated as the sum of the individual compound concentrations.

## 3. Results

### 3.1. Identification and Quantitation of Phytochemical Profile of Supercritical Extracts of Lemon Verbena by LC/MS

**Figure 1:**
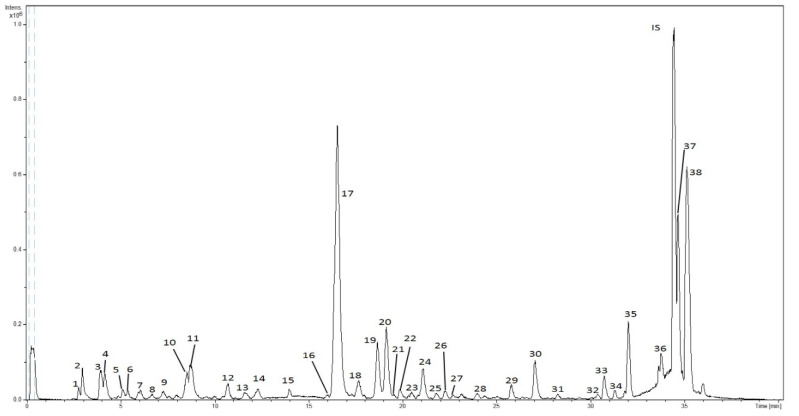
BPC of a supercritical extract of lemon verbena.

HPLC-ESI-TOF-MS analyses of SFE extracts were carried out to determine the potential application of SFE to recover phytochemicals from lemon verbena leaves. Figure 1 shows a representative base peak chromatogram (BPC) of SFE extracts. The numeration of the peaks was done according to their elution order (as compiled in Table 2). Furthermore, the calculated *m/z*, experimental *m/z*, molecular formula (M-H), mass error (ppm), isotopic pattern (mSigma) and proposed compound are also displayed.

**Figure 2:**
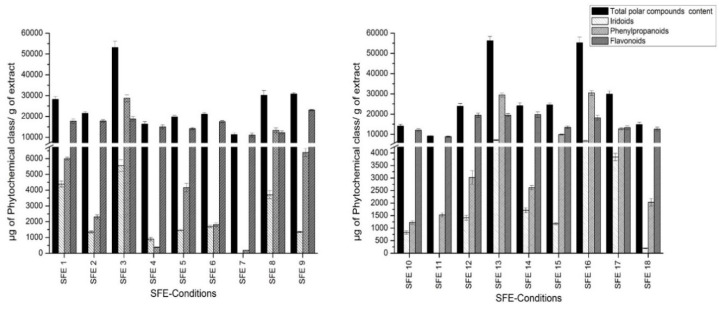
Quantitation of total polar content of obtained extracts.

The phenolic fraction consists of a heterogeneous mixture of compounds, which in most cases are not commercially available. Therefore, compounds which had no available commercial standards were tentatively quantitated using standards with similar structures. Phenylpropanoids were quantified using the verbascoside calibration curve. The loganic acid calibration curve was used to quantify iridoid glycosides, whereas flavonoids were quantified using the quercetin curve. Figure 2 shows the total polar content in SFE extracts for each chemical group calculated as a sum of the individual compound concentrations. The individual concentration of each compound is shown in Table S1.

A total of 38 compounds were detected in SFE extracts with the HPLC-ESI-TOF method. Of these compounds, 29 were identified by comparison of mass spectra and the available literature, and they were classified into six different groups corresponding to their chemical structures.

After examination of mass data provided by the analytical platform, a total of nine iridoid glycosides were found. According to their elution order, peaks 2 (shanzhiside), 3 (gardoside) and 7 (theveside) were previously identified in lemon verbena [14,19]. In addition, peak 9 was identified as myxopyroside, which was previously characterized in lemon verbena [17]. Moreover, compound 21 was identified as hydroxyl-campsiside, which was also detected in vegetable sources from the order Lamiales [20]. Compound 22 was tentatively identified as lippianoside B, which has also been found in *Aloysa triphylla* [21]. Compounds 23 and 34 were characterized as durantoside I and manuleoside, respectively [17]. Finally, compound 16, which displayed a retention time of 16.0 min and a molecular formula of C_26_H_32_O_14_, was identified as lamiidoside. This compound was tentatively characterized for the first time in lemon verbena, since it was previously identified in *Duranta erecta* (Lamiales) [22].

Considering the results of the iridoid quantification shown in Figure 2, the highest iridoid retrieval was provided by SFE condition 13 (40 °C, 400 bar, 15% co-solvent): 7172 µg of iridoids/g of extract. Condition 7 (60 °C, 150 bar, 7% co-solvent) was shown to be the worst in terms of recovering this chemical group. It is necessary to remark that gardoside was the most representative iridoid, and its concentration ranged from 3057 (SFE 3; 50 °C, 275 bar, 17% co-solvent) to 3136 µg of gardoside/g of extract (SFE 13). These results differ from the other outcomes reported for lemon verbena extractions, where theveside was the most abundant iridoid [17]. The rest of the iridoid glycosides showed greater variation depending on the condition applied.

In addition, the HPLC-ESI-TOF-MS platform provided information for ten phenylpropanoids. Indeed, the majority of the characterized phenolic compounds belonged to this chemical group. Compound 4 was characterized as verbasoside. Compounds 17, 19 and 20 displayed molecular formula allowing them to be identified according to a previously reported elution order as verbascoside, isoverbascoside and forsythoside A, respectively [23]. Verbascoside and its isomers are considered as the most representative phenylpropanoids in lemon verbena [17]. Moreover, compound 18 was also detected in the *Lippia* genus and was identified as lariciresinol glucopyranoside [21]. Compound 33 was determined to be osmanthisude B. In addition, two compounds were also detected that showed the same molecular formula (C_30_H_38_O_15_): leucoseptoside A (peak 24) and an isomer (peak 27). They were found in *Eremophila maculata,* a plant belonging to the order Lamiales [24]. Peaks 30 and 31 had the same *m/z* and similar retention times, and they were characterized as martynoside and its isomer, respectively [17].

As far as phenylpropanoid quantification is concerned, the range of retrieval was from 184 (SFE 7; 60 °C, 150 bar, 7% co-solvent) to 30,448 µg of phenylpropanoids/g of extract (SFE 16; 60 °C, 400bar, 15% co-solvent). Verbascoside was the most abundant compound, showing greater amounts in extracts SFE 3, 13 and 16 and reaching an amount of 21,984 µg of verbascoside/g of extract.

Overall, the applied method enabled the separation and identification of three different flavonoids, all of them eluted at the end of the analytical run. The first eluted compound was methyl-quercetin (peak 35). Furthermore, peak 37 was tentatively identified as dimethyl-kaempferol. The last flavonoid eluted from the column was dimethyl-quercetin (peak 38). All of them were previously described in *Lippia* [17]. These compounds were found in great amounts throughout the experiment, reaching values from 8921 to 23,113 µg of flavonoids/g of extract. In this sense, dimethyl quercetin was the most abundant flavonoid in SFE 9 (36 °C, 275 bar, 11% co-solvent), reaching the level of 15,622 µg of dimethyl quercetin/g of extract. The rest of flavonoids also yielded with positive results.

Regarding other chemical compounds, only two compounds were found within the oxylipin group. Thus, compound 10 yielded a deprotonated molecular formula of C_18_H_27_O_9_ and was characterized as tuberonic acid glucoside according to the literature (Quirantes-Piné et al., 2013). Besides, peak 14 was characterized as its aglycone form, tuberonic acid.

Peak 1 gave an *m/z* at 341.1089 and a deprotonated molecular formula of C_12_H_21_O_11_. This compound was previously detected in lemon verbena as a disaccharide [25]. Peaks 5 and 6 presented the same *m/z* at 203.0925, and they were identified as dihydroharpagenin isomers. This association was made on the basis that these compounds were previously detected in plants from the order Lamiales [26]. Furthermore, a novel compound was tentatively identified in lemon verbena and related to peak 11. The MS spectra generated a molecular formula (C_19_H_31_O_8_) enabling its characterization as hydroxyl-epoxy-ionol-glucopyranoside. This compound was previously found in *Isodon japonicus* (Lamiaceae family) [27]. Finally, a primeverin derivative was found and associated to peak 29. It was characterized as octen-primeveroside.

It is necessary to remark that this extraction technique provided a lower variety of compounds from lemon verbena compared to previous reports which applied different extraction methods to retrieve bioactive compounds from lemon verbena. Nevertheless, this extraction method allowed the recovery of up to six different compounds which could not be extracted from lemon verbena using other techniques. Moreover, extracts were obtained that had greater concentrations of methylated flavonoids than those from other extraction techniques performed on this botanical source [17,18]. Hence, SFE offered higher selectivity in the extraction of some compounds.

### 3.2. Effect of Independent Variables on Extraction Yield and Bioactive Compound Recovery

**Figure 3:**
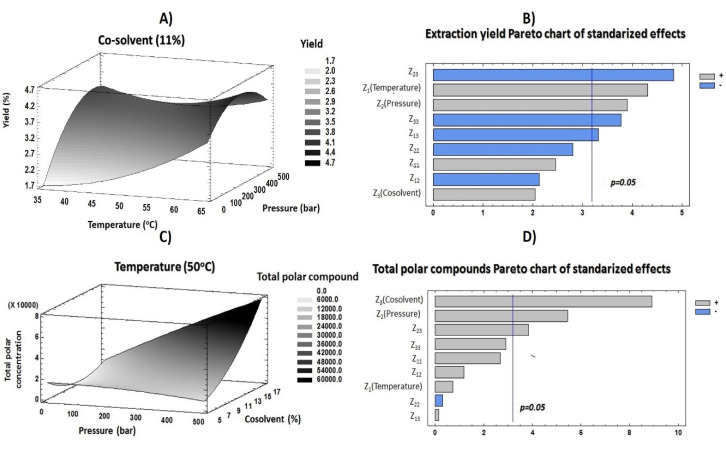
Response surface methodology (RSM) (**A**,**C**) and Pareto (**B**,**D**) plots of response variables evaluated.

With the purpose of evaluating the effects of each factor (temperature, pressure and co-solvent percentage) on the response variables (yield and total polar compound contents), an RSM based on a 2^3^ CCD was developed. Table 3 compiles the influence of each factor, and Figure 3B,D includes the obtained Pareto plots for each response variable. These plots are tools that provide graphical information to understand the response variable behavior and the influence of each independent variable at a 95% confidence interval.

Temperature is a determining factor since higher values generate variation in supercritical fluid density and consequently in its penetration capability. Furthermore, the solubility of specific compounds is increased when temperature is raised [28]. Both scenarios enabled the retrieval of compounds from raw material. Therefore, at constant pressure, an increase of temperature produces a decrease of supercritical fluid density and consequently improves the compounds’ solubility [29].

Concerning extraction yield, temperature showed a significant positive effect on this response (Figure 3B). These results indicated that a high temperature caused a rupture of plant cells and enabled all components in them (both desirable and nondesirable compounds) to be released. Moreover, the low density of solvents at a high temperature allowed better wetting of leaves and therefore enabled the transfer of heat into the sample. All of this contributed to a great retrieval of a wide variety of compounds, increasing the amount of extracts recovered after supercritical procedures. Although high temperatures may degrade some thermosensitive compounds [30,31], the results in Figure 3D and Table 3 show that temperature had a positive influence on the recovery of polar compounds. These results may be due to the different structures of compounds and the raw material. However, this factor did not present any significant values. According to other studies, the decrease of density caused by high temperatures could enable the penetration of solvents into the matrix, improving the solubility of polar compounds into the solvent mix [11,29].

On the other hand, extraction yield and pressure were positively correlated (Figure 3B). The results revealed that higher pressures produced an improvement of extraction yield according to CO_2_ and co-solvent densities, which were increased at higher pressures, resulting in greater solvation power and, consequently, an increased recovery of compound contents in the sample. The sum of all components extracted from the plant generated an increase in the quantity of attained extract. Pressure also showed a positive effect on the total polar compound content. In previous studies, it has been reported that greater recoveries of phenolic compounds from plants were achieved at high pressure values [32]. This could be related to the effect of great pressures on solvents, such as CO_2_ and ethanol. Indeed, high pressure values generate an increase in solvent and co-solvent densities; consequently, this fact modifies solute solubility [33].

The percentage of co-solvent added as modifier causes a polarity adjustment that is necessary to achieve great recoveries of phenolics due to their hydrophilic character. As far as extraction yield is concerned, co-solvent exerted a significant negative influence combined with both pressure and temperature. According to previous reports, high concentrations of ethanol added into the solvent mix may hinder the retrieval of components from matrix. This effect could be related to the reduction of the extraction mixture homogeneity [29]. Nevertheless, ethanol was needed to improve the solubility of polar compounds [28]. Moreover, the addition of an organic co-solvent such as ethanol improves the solvating power of supercritical fluids [34]. Therefore, an increase of co-solvent concentration could provide a greater retrieval of polar compounds. Indeed, this independent factor had the most influential positive effect on polar compound content. Furthermore, the interaction between this factor and pressure also had significant effects. These outcomes could indicate that co-solvent addition reduced the supercritical fluid polarity, increasing the solubility of polar compounds. Meanwhile, pressure contributed in improving the solubility of phytochemicals.

### 3.3. Evaluation of Model Fitting Parameters

The evaluation of the influence of each factor on the response variables enabled the establishment of the optimal conditions and the maximization of extraction yield and the phytochemical recovery from lemon verbena. This goal was achieved using several fitting parameters which were assessed in order to determine the model adequacy. ANOVA test results are shown in Table 4. Both responses displayed a good homogeneity of the experimental data, as revealed their CV. In this sense, total polar compound content presented 10.42%, whereas extraction yield presented 2.57%. Moreover, an accurate prediction of response behavior was reached with the proposed experimental model (R^2^) (Table 5). Indeed, 90% of the obtained results could be predicted with this experimental model. Moreover, to evaluate the model adequacy, two parameters were also evaluated. Firstly, lack-of-fit revealed an acceptable adequacy of the proposed model, since both responses revealed nonsignificant values (*p* > 0.05). Lastly, model adequacy showed a great adjustment to total polar compound content response. Unfortunately, extraction yield did not have a good fitting, since the *p*-value was nonsignificant. The data were statistically evaluated by implementing response surface models (Figure 3A,C) which enabled the determination of the region of extraction factors that would maximize the variable responses. Moreover, the coefficients displayed in Table 3 and the experimental results shown in Table 5 support these optimal conditions.

#### 3.3.1. Extraction Yield Optimization

Regarding extraction yield, the obtained values ranged from 2.028 to 4.683%. Although SFE may provide a selective extraction of bioactive compounds, the efficiency of the process was much lower than other techniques such as pressurized liquid extraction and microwave-assisted extraction [17,18] applied on lemon verbena. However, these results were similar to those obtained by SFE procedure performed on *Hibiscus sabdariffa* or *Lavandula angustifolia* [31,35] but better than those of the SFE of *Castanea sativa* shells [36]. Considering Table 5, the best condition to attain greater yields was SFE 17 (64 °C, 275 bar, 11% co-solvent). Equation (3) summarizes a relationship between the extraction yield and significant independent variables:

(3) $$Y=-5.620+0.088\;Z_{1}+0.009\;Z_{2}-0.016\;Z_{3}^{2}-0.006\;Z_{1}Z_{3}-0.001\;Z_{2}Z_{3}\;$$

These results revealed that the linear effect of temperature contributed in a greater measure than the single effect of pressure. Concerning the interaction between factors and according to Figure 3B, the interaction between pressure and co-solvent contributed more than the interaction between temperature and co-solvent. Figure 3A shows the extraction yield behavior according to the two most influential factors (temperature and pressure). In this plot, it can be seen that the maximum extraction yield was achieved when medium pressure and high temperature were applied. These results supported the establishment of optimum conditions at 64 °C, 379 bar and 7% co-solvent. The model resulted in a predictable yield value of 4.928%, which was higher than the experimental result obtained in condition 17 (4.299%).

#### 3.3.2. Total Polar Compound Content Optimization

Table 5 reports the total polar compound contents obtained at each experimental point. Overall, the values ranged from 9182 to 56,279 µg of polar compound/g of dried extract. Conditions 13 (40 °C, 400 bar, 15% co-solvent), 16 (60 °C, 400 bar, 15% co-solvent) and 3 (50 °C, 275 bar, 17% co-solvent) enabled a great retrieval, upwards of 50,000 µg of polar compound/g of dried extract, whereas SFE 11 (50 °C, 275 bar, 5% co-solvent) yielded the lowest polar compound recovery.

After examining the results of the experimental design and analyzing the effect of each factor and its combinatory effects on the total polar compound content, this response variable was fitted in a simplified quadratic equation that includes only the significant factors (Equation (4)):

(4) $$Y=16174.300-81.211\;Z_{2}-488.563\;Z_{3}+12.713\;Z_{2}Z_{3}\;$$

As mentioned above, pressure and percentage of ethanol were the most influential factors on polar compound extraction. This behavior was similar to that previously reported by Espinosa-Pardo and Roselló-Soto [11,36]. According to the Figure 3D and this reduced model, the percentage of co-solvent was the main determinant in increasing the retrieval of compounds.

The good adjustment of this response enabled optimal conditions for maximizing this response to be discerned. In this sense, Figure 3C displays the total polar compound content. This plot shows the effect of pressure and co-solvent at constant temperature on the selected response variable and provides the optimal values at 57 °C, 451 bar and 17% co-solvent. Here, the pressure was the highest value that the supercritical fluid equipment allowed, whereas the percentage of ethanol cannot be higher because it would not assure the supercritical state of CO_2_ throughout the proposed conditions in the experimental design. The predicted result obtained after applying these optimum conditions was 73,982 µg of polar compound/g of dried extract. This value was considerably greater than that obtained in SFE 13, which showed the highest polar compound recovery.

## 4. Conclusions

In this study, a new experimental design was created with the purpose of obtaining high-quality phenolic extracts to develop functional food. The dependent variables of total phenolic content and yield were successfully optimized. The fitting parameters of the proposed model indicated that the second-order polynomial model gave a mathematical explanation of the SFE procedures. Indeed, the proposed model allowed an accurate prediction of the studied response variables and enabled the effects of each factor during SFE processing to be discerned. In this sense, the percentage of co-solvent was the determinant in increasing the retrieval of compounds, whereas temperature was revealed to be an important parameter in increasing extraction yield. The individual action of pressure or its interaction with other factors enabled the maximization of each response. Particularly, flavonoids were recovered in higher concentrations, whereas iridoid glycoside retrieval was lower. Finally, verbascoside was the most recovered phenylpropanoid. These results, compared with previous reports, revealed that SFE allowed the extraction of six new compounds in lemon verbena and enabled higher recoveries of some compounds, such as methylated flavonoids, than those of other advanced extraction methods, enhancing the obtainment of enriched extracts in specific chemical groups. It should be considered that the use of the SFE process provided a more selective phytochemical extraction from lemon verbena, as well as the retrieval of new compounds. Therefore, the evaluation of the impact of the independent variables provides useful information for the industrial process scale-up of the extraction of lemon verbena functional ingredients.

## Figures and Tables

**Table 1:** Experimental values of central composite design (CCD) factors.

Symbols	Factors	Coded Levels				
		−α	−1	0	+1	+α
		Experimental Levels				
**Z1**	Temperature (°C)	36	40	50	60	64
**Z2**	Pressure (bar)	98	150	275	400	452
**Z3**	Co-solvent (% ethanol)	5	7	11	15	17

**Table 2:** Polar composition of supercritical extracts of lemon verbena.

Peak	RT (min)	Proposed Compound	*m/z*	*m/z Exp*	Molecular Formula	Error (ppm)	mSigma
**Iridoid glycosides**							
2	3.0	Shanzhiside	391.1224	391.1246	C_16_H_23_O_11_	5.6	27.2
3	4.0	Gardoside	373.1142	373.1140	C_16_H_21_O_10_	−0.6	57.9
7	6.0	Theveside	389.1097	389.1089	C_16_H_21_O_11_	−2.0	13.3
9	7.3	Myxopyroside	449.1286	449.1301	C_18_H_25_O_13_	3.3	9.4
16	16.0	Lamiidoside	567.1719	567.1687	C_26_H_31_O_14_	5.7	11.4
21	19.6	Hydroxy-campsiside	521.1628	521.1664	C_25_H_29_O_12_	7.0	44.2
22	19.9	Lippianoside B	549.1608	549.1614	C_26_H_29_O_13_	1.0	16.4
23	20.5	Durantoside I	551.1769	551.1770	C_26_H_31_O_13_	1.0	4.5
34	31.2	Manuleoside H	569.2287	569.2240	C_27_H_37_O_13_	6.1	9.9
**Phenylpropanoids/phenylethanoids**							
4	4.2	Verbasoside	461.1676	461.1664	C_20_H_29_O_12_	3.9	7.3
17	16.5	Verbascoside	623.2039	623.1981	C_29_H_35_O_15_	−1.6	2.9
18	17.7	Lariciresinol glucopyranoside	521.2031	521.2028	C_26_H_33_O_11_	4.4	28.8
19	18.7	Isoverbascoside	623.1984	623.1981	C_29_H_35_O_15_	−0.5	12.2
20	19.1	Forsythoside A	623.1990	623.1981	C_29_H_35_O_15_	−1.3	3.1
24	21.1	Leucoseptoside A or isomer	637.2132	637.2138	C_30_H_37_O_15_	1.3	5.7
27	22.6	Leucoseptoside A or isomer	637.2138	637.2158	C_30_H_37_O_15_	−3.2	12.8
30	27.0	Martynoside or isomer	651.2327	651.2294	C_31_H_39_O_15_	0.6	16.1
31	28.3	Martynoside or isomer	651.2327	651.2307	C_31_H_39_O_15_	−2.0	19.3
33	30.7	Osmanthisude B	591.2135	591.2083	C_29_H_35_O_13_	−8.8	17.3
**Flavonoids**							
35	32.0	Methyl-quercetin	315.0540	315.0510	C_16_H_11_O_7_	−4.1	11.9
37	34.6	Dimethyl-kaempferol	299.0539	299.0561	C_16_H_11_O_6_	7.5	3.8
38	35.1	Dimethyl-quercetin	329.0652	329.0667	C_17_H_13_O_7_	4.4	3.6
**Oxylipins**							
10	8.5	Tuberonic acid glucoside	387.1663	387.1661	C_18_H_27_O_9_	−0.7	4.5
14	12.3	Tuberonic acid	225.1143	225.1132	C_12_H_17_O_4_	4.7	31.8
**Sugars**							
1	2.8	Disaccharid*e*	341.1089	341.1089	C_12_H_21_O_11_	0.1	29.3
**Other Compounds**							
5	5.1	Dihydroharpagenin or isomer	203.0916	203.0925	C_9_H_15_O_5_	4.2	3.8
6	5.4	Dihydroharpagenin or isomer	203.0924	203.0925	C_9_H_15_O_5_	0.4	15.7
11	8.7	Hydroxy-epoxy-ionol glucopyranoside	387.1943	387.1966	C_19_H_31_O_8_	9.4	7.3
29	25.8	Octen-primeveroside	421.2051	421.2079	C_19_H_33_O_10_	6.6	14.2
**Unknown**							
8	6.7	UK1	201.1123	201.1132	C_10_H_17_O_4_	4.4	10.5
12	10.7	UK2	435.2181	435.2236	C_27_H_31_O_5_	−0.9	49.8
13	11.6	UK2 derivative	433.2070	433.2079	C_27_H_29_O_5_	−2.6	55.3
15	14.0	UK3	201.1114	201.1132	C_10_H_17_O_4_	5.5	18.9
25	21.8	UK4	417.2105	417.2130	C_20_H_33_O_9_	5.6	8.4
26	22.2	UK5	187.0973	187.0976	C_9_H_15_O_4_	5.7	10.1
28	23.9	UK6	183.1027	183.1034	C _10_H_15_O_3_	−4.1	14.6
32	30.3	UK7	375.2049	375.2024	C_18_H_31_O_8_	0.4	14.0
36	33.7	Uk8	327.2202	327.2177	C_18_H_31_O_5_	1.1	0.3

**Table 3:** Equation coefficients and *p*-Value for the independent variables (β_0_, independent constant, β_1_, Temperature; β_2_, Pressure; β_3_, Co-solvent).

Factors	Total Polar Content		Yield	
	Coefficient	*p*-Value	Coefficient	*p*-Value
β_0_	174,734.000		−3.910	
β_1_	−4819.360	0.523	−0.050	*0.023
β_2_	−141.311	*0.012	0.022	*0.030
β_3_	−7406.600	*0.003	0.863	0.133
β_1_·β_1_	44.199	0.075	0.002	0.091
β_2_·β_2_	−0.033	0.772	−0.000	0.067
β_3_·β_3_	301.068	0.062	−0.016	*0.032
β_1_·β_2_	1.570	0.320	−0.000	0.123
β_1_·β_3_	5.891	0.895	−0.006	*0.045
β_2_·β_3_	12.713	*0.031	−0.016	*0.017

* Significant coefficients (*p <* 0.050).

**Table 4:** Fitting parameters for the full model.

	Total Polar					Yield				
	***Sum of Squares***	***Degrees of Freedom***	***Mean Squares***	***Fisher-Ratio***	***p-Value***	***Sum of Squares***	***Degrees of Freedom***	*Mean Squares*	*Fisher Ratio*	*p-Value*
**Model**	2.393E+09	3	7.976E+08	10.014	<0.001 *	1.468	3	0.489	2.187	0.135
**Residual**	1.115E+09	14	7.965E+07			3.133	14	0.224		
**Lack-of-fit**	3.510E+08	5	7.020E+07	3.220	0.182	0.426	5	0.0851	2.200	0.274
**Pure error**	6.540E+07	3	2.180E+07			0.116	3	0.0387		
**Total (corr.)**	3.510E+09	17				4.601	17			
**R^2^**	0.881					0.882				
**CV**	10.42					2.57				

* Significant coefficients (*p <* 0.050).

**Table 5:** Experimental and predicted data for the response variables obtained from the CCD.

Experimental Point	*Z_1_*	*Z_2_*	*Z_3_*	Extraction Yield		Total Content *	
				*Predicted*	*Observed*	*Predicted*	*Observed*
1	60	150	15	3.717	3.692	29,319	28,213 ± 1381
2	50	275	11	3.612	3.582	20,766	21,521 ± 821
3	50	275	17	3.251	3.207	47,414	53,152 ± 3111
4	50	98	11	2.908	2.945	10,401	16,348 ± 1138
5	40	150	15	3.393	3.448	27,376	19,799 ± 832
6	40	150	7	2.028	2.159	16,029	21,069 ± 700
7	60	150	7	3.275	3.027	17,971	11,279 ± 988
8	60	400	7	4.093	3.978	19,917	30,183 ± 2312
9	36	275	11	3.607	3.343	28,232	30,830 ± 639
10	50	275	11	3.612	3.521	20,766	14,125 ± 848
11	50	275	5.3	2.922	3.086	13,387	9182 ± 352
12	50	275	11	3.612	3.847	20,766	23,895 ± 1384
13	40	400	15	3.461	3.648	54,748	56,279 ± 2096
14	50	275	11	3.612	3.377	20,766	24,085 ± 1572
15	50	452	11	3.535	3.618	31,131	24,626 ± 828
16	60	400	15	3.193	3.002	56,691	55,282 ± 2760
17	64	275	11	4.299	4.683	30,980	29,915 ± 1552
18	40	400	7	3.439	3.404	17,974	14,861 ± 1163

* Concentrations are expressed as µg of analyte/g dried extract; Z_1_ represents temperature (°C), Z_2_ represents pressure (bar) and Z_3_ represents co-solvent percentage.

## Supplementary Materials

The following are available online at https://www.mdpi.com/2304-8158/9/7/931/s1, Table S1: Quantitation of individual compounds presents in *L. citriodora* supercritical extracts (µg of analyte/g of dried extract). Value = X ± SD.
