# Supplementary material for: Optimized Extraction of Phenylpropanoids and Flavonoids from Lemon Verbena Leaves by Supercritical Fluid System Using Response Surface Methodology
Source: Foods. 2020 Jul 14;9(7):931. doi: 10.3390/foods9070931 (PMC7404463; doi:10.3390/foods9070931)
Supplement: Supplementary file 1 [file foods-09-00931-s001.zip › foods-836708-supplementary.pdf]

foods

Supercritical-Fluid Process Control to Functional Food Ingredient Development: *Lippia citriodora*

Francisco-Javier Leyva-Jiménez[1], Jesús Lozano-Sánchez[1,2]*, Maria de la Luz Cádiz-Gurrea[1,3], Alvaro Fernández-Ochoa[1,3], David Arráez-Román[1,3+], Antonio Segura-Carretero[1,3+].

[1] Functional Food Research and Development Center, Health Science Technological Park, Avenida del Conocimiento s/n, E-18016 Granada, Spain [2]; FJLJ: jleyva@cidaf.es

[2] Department of Food Science and Nutrition , University of Granada, Campus of Cartuja, 18071 Granada, Spain; JLS: jesusls@ugr.es

[3] Department of Analytical Chemistry, Faculty of Sciences, University of Granada, Fuentenueva s/n, E- 18071 Granada, Spain; MdlLCG: mluzcadiz@ugr.es; AFO: alvaroferochoa@ugr.es; DAR: darraez@ugr.es; ASC: ansegura@ugr.es.

[+] These authors are joint senior authors on this work
* Correspondence: jesusls@ugr.es; Tel.: +34-958-637083

Received: 31 May 2020; Accepted: 6 July 2020; Published: 14 July 2020

Supporting information Table 1A : Quantitation of individual compounds presents in *L.* *citriodora* supercritical extracts (µg of analyte/ g of dried extract). Value = X ± SD.

	SFE 1	SFE 2	SFE 3	SFE 4	SFE 5	SFE 6	SFE 7	SFE 8	SFE 9
Iridioids	***4388 ± 185***	***1345 ± 68***	***5558 ± 369***	***899 ± 108***	***1460 ± 49***	***1706 ± 79***	***NQ***	***3708 ± 255***	***1357 ± 35***
Shanzhiside	98 ± 19	NQ	240 ± 2	NQ	NQ	NQ	ND	NQ	NQ
Gardoside	2490 ± 71	805 ± 28	3057 ± 145	719 ± 57	1351 ± 37	1397 ± 34	NQ	2086 ± 78	1171 ± 3
Theveside	357 ± 22	NQ	475 ± 33	NQ	NQ	NQ	ND	111 ± 33	NQ
Myxopyroside	720 ± 2	433 ± 14	324 ± 41	180 ± 51	74 ± 4	268 ± 39	NQ	721 ± 74	181 ± 35
Lamiidoside	NQ	97 ± 23	215 ± 14	ND	NQ	NQ	ND	NQ	NQ
Hydroxycampsiside	21 ± 14	NQ	239 ± 52	NQ	NQ	NQ	ND	49 ± 22	NQ
Lippianoside B	39 ± 2	NQ	248 ± 35	ND	NQ	NQ	ND	160 ± 17	NQ
Durantoside I	219 ± 41	NQ	467 ± 46	NQ	24 ± 3	NQ	ND	253 ± 18	NQ
Manuleoside H	429 ± 14	40 ± 3	293 ± 1	NQ	11 ± 5	41 ± 6	ND	328 ± 13	4.9 ± 0.4
Phenylpropanoids	**5992 ± 97**	**2312 ± 136**	**28745 ± 1617**	**388 ± 20**	**4167 ± 265**	**1816 ± 121**	**184 ± 5**	**13318 ± 1216**	**6390 ± 249**
Verbasoside	384 ± 21	NQ	655 ± 54	NQ	109 ± 9	12 ± 5	NQ	306 ± 28	61 ± 10
Verbascoside	3629 ± 30	1949 ± 99	21984 ± 1200	388 ± 20	3247 ± 214	1560 ± 73	184 ± 5	9764 ± 1042	5210 ± 195
Lariciresinol glucopyranoside	478 ± 11	147 ± 13	490 ± 39	NQ	133 ± 4	105 ± 16	NQ	449 ± 24	117 ± 13
Isoverbascoside	141 ± 8	NQ	1389 ± 129	NQ	81 ± 10	NQ	NQ	644.5 ± 0.1	207 ± 7
Forsythoside A	249 ± 7	NQ	1829 ± 107	NQ	144.1 ± 0.1	NQ	NQ	611 ± 18	366 ± 9
Leucoseptoside A or isomer	176 ± 2	NQ	990 ± 31	NQ	104 ± 8	NQ	NQ	445 ± 30	119 ± 1
Leucoseptoside A or isomer	NQ	NQ	NQ	ND	NQ	NQ	ND	NQ	NQ
Martynoside or isomer	604 ± 18	187 ± 14	974 ± 56	NQ	244 ± 14	107 ± 17	NQ	806 ± 46	255 ± 8
Martynoside or isomer	NQ	NQ	NQ	NQ	NQ	NQ	ND	NQ	NQ
Osmanthisude B	331.0 ± 0.6	29 ± 10	434 ± 1	NQ	105 ± 6	32 ± 10	NQ	293 ± 28	55 ± 6
Flavonoids	**17833 ± 1099**	**17864 ± 617**	**18849 ± 1128**	**15061 ± 1010**	**14172 ± 518**	**17547 ± 500**	**11095 ± 983**	**12274 ± 841**	**23113 ± 355**
Methyl quercetin	929 ± 63	958 ± 29	1111 ± 96	889 ± 52	885 ± 45	1117 ± 30	140 ± 9	718 ± 53	1044 ± 86
Dimethyl Kaempferol	5028 ± 285	4605 ± 59	5162 ± 147	3625 ± 124	3837 ± 261	4801 ± 114	1930 ± 156	3143 ± 18	6447 ± 48
Dimethyl quercetin	11876 ± 751	12301 ± 529	12576 ± 885	10547 ± 834	9450 ± 212	11629 ± 356	9025 ± 818	8413 ± 770	15622 ± 221
Total	***28213 ± 1381***	***21521 ± 821***	***53152 ± 3111***	***16348 ± 1138***	***19799 ± 832***	***21069 ± 700***	***11279 ± 988***	***30183 ± 2312***	***30830 ± 639***

*NQ: Not quantified. Compound detected, but their concentration is between the detection and quantification limits. *ND: Not detected. Compound concentration is below of detection limit.

Supporting information Table 1B : Quantitation of individual compounds presents in *L. citriodora* supercritical extracts (µg of analyte/ g of extract). Value = X ± SD.

Condition<br>Compound	SFE 10	SFE 11	SFE 12	SFE 13	SFE 14	SFE 15	SFE 16	SFE 17	SFE 18
Iridioids	**825 ± 60**	**NQ**	**1416 ± 101**	**7172 ± 215**	**1715 ± 101**	**1173 ± 40**	**6663 ± 436**	**3840 ± 144**	**199 ± 15**
Shanzhiside	NQ	ND	NQ	265 ± 25	NQ	NQ	147.8 ± 0.3	NQ	NQ
Gardoside	395 ± 15	NQ	976 ± 71	3136 ± 26	1019 ± 58	568 ± 34	2904 ± 188	1671 ± 2	19 ± 6
Theveside	NQ	ND	NQ	676 ± 11	NQ	NQ	505 ± 41	87 ± 17	NQ
Myxopyroside	408 ± 32	NQ	414 ± 24	680 ± 29	316 ± 10	574 ± 5	834 ± 59	1018 ± 22	180 ± 9
Lamiidoside	NQ	ND	NQ	326 ± 15	380 ± 33	NQ	299 ± 29	NQ	ND
Hydroxycampsiside	NQ	ND	NQ	354 ± 30	NQ	NQ	352 ± 50	21 ± 6	NQ
Lippianoside B	NQ	ND	NQ	503 ± 11	NQ	NQ	438 ± 25	187 ± 56	NQ
Durantoside I	NQ	ND	NQ	706 ± 20	NQ	NQ	619 ± 17	288 ± 18	NQ
Manuleoside H	22 ± 13	ND	26 ± 6	526 ± 48	NQ	31 ± 1	564 ± 27	568 ± 23	NQ
Phenylpropanoids	**1230 ± 72**	**1531 ± 77**	**3027 ± 269**	**29429 ± 1027**	**2620 ± 86**	**9943 ± 192**	**30448 ± 1118**	**12739 ± 491**	**2034 ± 148**
Verbasoside	NQ	NQ	24.5 ± 0.7	747 ± 19	3.8 ± 0.1	NQ	641 ± 57	332 ± 19	NQ
Verbascoside	1079 ± 65	1467 ± 70	2489 ± 234	21847 ± 801	2219 ± 71	8537 ± 128	21908 ± 752	8631 ± 391	2034 ± 148
Lariciresinol glucopyranoside	56 ± 2	NQ	197 ± 6	734 ± 24	126 ± 0.6	99 ± 6	690 ± 24	562 ± 23	NQ
Isoverbascoside	NQ	NQ	13 ± 4	1837 ± 63	NQ	564 ± 13	1896 ± 17	737 ± 2	NQ
Forsythoside A	NQ	64 ± 7	9.6 ± 0.5	1267 ± 69	45 ± 3	94 ± 9	2415 ± 122	464 ± 46	NQ
Leucoseptoside A or isomer	NQ	NQ	5 ± 1	1078 ± 11	6 ± 1	215 ± 14	1058 ± 71	496 ± 0.4	NQ
Leucoseptoside A or isomer	NQ	NQ	NQ	NQ	NQ	NQ	NQ	NQ	ND
Martynoside or isomer	95 ± 5	NQ	249 ± 21	1301 ± 24	204 ± 9	355 ± 19	1237 ± 50	1070 ± 3	NQ
Martynoside or isomer	NQ	ND	NQ	NQ	NQ	NQ	NQ	NQ	NQ
Osmanthisude B	NQ	NQ	40 ± 2	618 ± 16	16 ± 1	79 ± 3	603 ± 27	447 ± 7	NQ
Flavonoids	**12070 ± 716**	**8921 ± 275**	**19452 ± 1014**	**19675 ± 854**	**19750 ± 1385**	**13510 ± 596**	**18171 ± 1206**	**13336 ± 917**	**12628 ± 1000**
Methyl quercetin	626 ± 21	119 ± 10	1164 ± 59	1386 ± 19	1005 ± 45	502 ± 13	1588 ± 52	986 ± 97	617 ± 48
Dimethyl Kaempferol	2911 ± 182	1417 ± 89	5152 ± 247	5262 ± 316	5488 ± 124	3083 ± 295	5101 ± 373	3006 ± 63	2612 ± 110
Dimethyl quercetin	8533 ± 513	7385 ± 176	13136 ± 708	13027 ± 519	13257 ± 1216	9925 ± 288	11482 ± 781	9344 ± 757	9399 ± 842
Total	***14125 ± 848***	***9182 ± 352***	***23895 ± 1384***	***56279 ± 2096***	***24085 ± 1572***	***24626 ± 828***	***55282 ± 2760***	***29915 ± 1552***	***14861 ± 1163***

*NQ: Not quantified. Compound detected, but their concentration is between the detection and quantification limits. *ND: Not detected. Compound concentration is below of detection limit.

© 2020 by the authors. Submitted for possible open access publication under the terms and conditions of the Creative Commons Attribution (CC BY) license (http://creativecommons.org/licenses/by/4.0/).
